# Supplementary material for: Mosaic patterns of selection in genomic regions associated with diverse human traits
Source: PLoS Genet. 2022 Nov 7;18(11):e1010494. doi: 10.1371/journal.pgen.1010494 (PMC9671423; doi:10.1371/journal.pgen.1010494)
Supplement: S1 Table — Number of traits (“# Traits”) in the full Evolutionary Atlas (top) and BOLT-LMM subset (bottom) with statistically significant enrichment for evolutionary measures (rows). Note, only traits with 50 or more associated regions are analyzed within the Evolutionary Atlas. The proportion out of all traits analyzed (“Proportion of All Traits (%)”) are shown for the Evolutionary Atlas (n = 290 traits) and BOLT-LMM set (n = 47 traits). Depletion refers to negative enrichment. (DOCX) [file pgen.1010494.s001.docx]

|  | **Evolutionary Atlas** | |  |  |
| --- | --- | --- | --- | --- |
| **Annotation** | # Traits | Proportion of  All Traits (%) |  |  |
| ARGweaver | 166 | 57.2 |  |  |
| Beta Score | 147 | 50.7 |  |  |
| LINSIGHT | 278 | 95.9 |  |  |
| PhastCons | 281 | 96.9 |  |  |
| PhyloP | 222 | 76.6 |  |  |
| F_ST_ afr-eas | 152 | 52.4 |  |  |
| F_ST_ afr-eur | 194 | 66.9 |  |  |
| F_ST_ eas-eur | 175 | 60.3 |  |  |
| XP-EHH afr-eas | 37 | 12.8 |  |  |
| XP-EHH afr-eur | 138 | 47.6 |  |  |
| XP-EHH eas-eur | 87 | 30 |  |  |
|  |  |  |  |  |
|  | **BOLT-LMM Set** | | | |
| **Annotation** | # Traits | Proportion of  All Traits (%) | Enrichment (# Traits) | Depletion (# Traits) |
| ARGweaver | 29 | 61.7 | 0 | 30 |
| Beta Score | 32 | 68.1 | 0 | 32 |
| LINSIGHT | 44 | 93.6 | 45 | 0 |
| PhastCons | 47 | 100 | 47 | 0 |
| PhyloP | 39 | 83 | 40 | 0 |
| F_ST_ afr-eas | 31 | 66 | 32 | 0 |
| F_ST_ afr-eur | 35 | 74.5 | 37 | 0 |
| F_ST_ eas-eur | 35 | 74.5 | 35 | 0 |
| XP-EHH afr-eas | 15 | 31.9 | 5 | 2 |
| XP-EHH afr-eur | 29 | 61.7 | 24 | 5 |
| XP-EHH eas-eur | 19 | 40.4 | 13 | 4 |
