# Supplementary figures and images for: Mosaic patterns of selection in genomic regions associated with diverse human traits
Source: PLoS Genet. 2022 Nov 7;18(11):e1010494. doi: 10.1371/journal.pgen.1010494 (PMC9671423; doi:10.1371/journal.pgen.1010494)

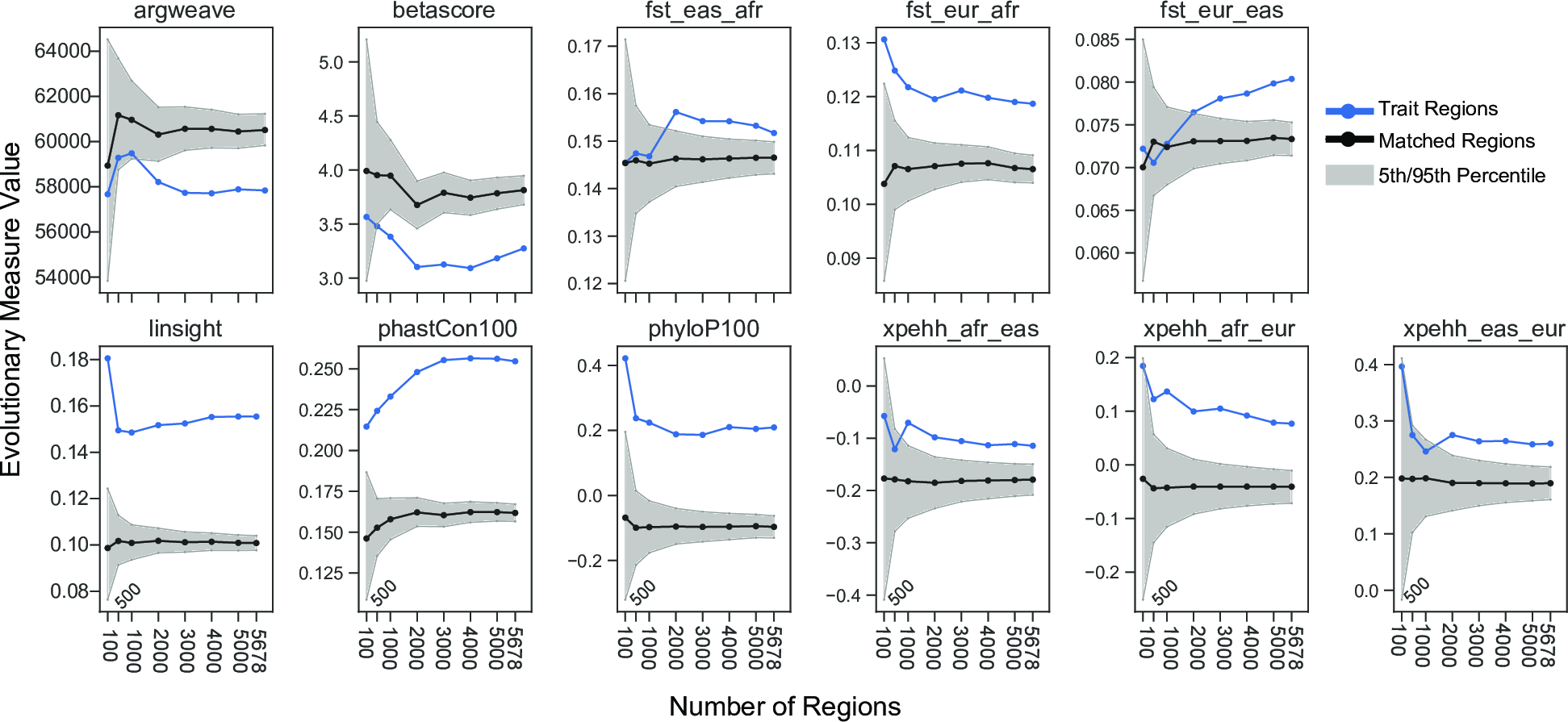

Supplement: S1 Fig — Using the Loh et. al. GWAS, we randomly undersampled the number of trait-associated regions without replacement (x-axis) and measured the mean evolutionary measure at trait-associated regions (blue line) and the matched background (mean: black line, gray shading between 5th and 95th percentiles). The observed evolutionary measures for trait-associated regions and their relative values compared to the matched background regions are consistent across different numbers of associated loci considered. (TIF) [file pgen.1010494.s002.tif]

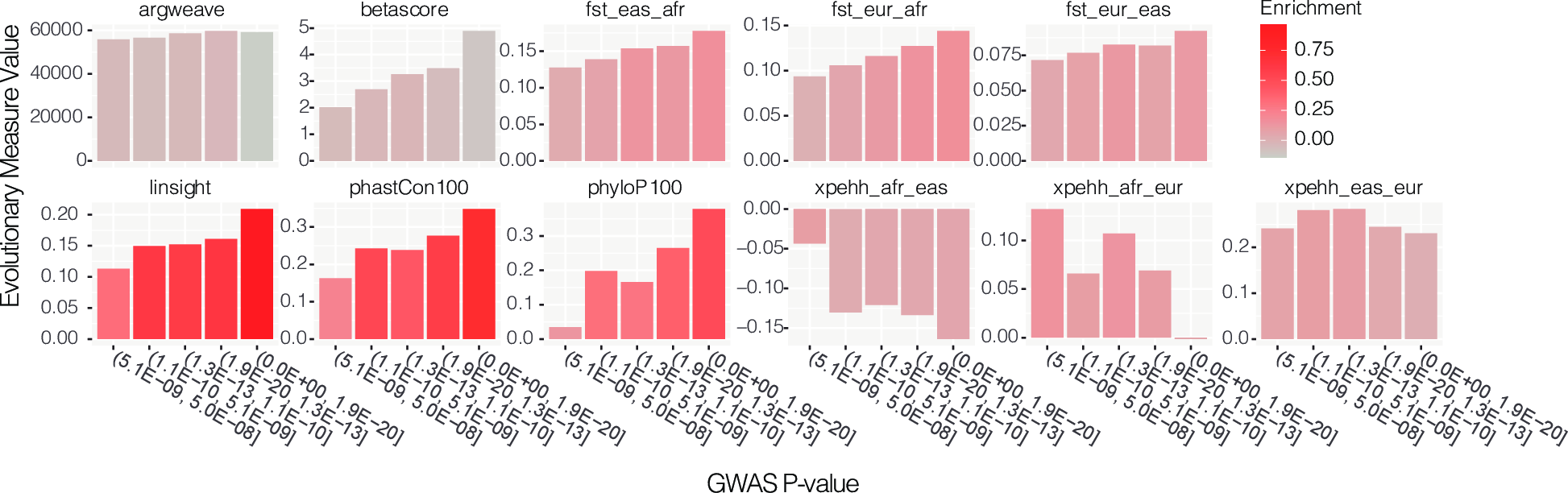

Supplement: S2 Fig — Using the Loh-2018 (Fig 2) GWAS, we partitioned trait-associated regions into five bins with equal number of regions based on GWAS p-value of the lead SNP in each region. Each plot represents the mean trait value (y-axis) for an evolutionary measure and each bar is colored by the evolutionary enrichment which is calculated as described in Fig 1D. (TIF) [file pgen.1010494.s003.tif]

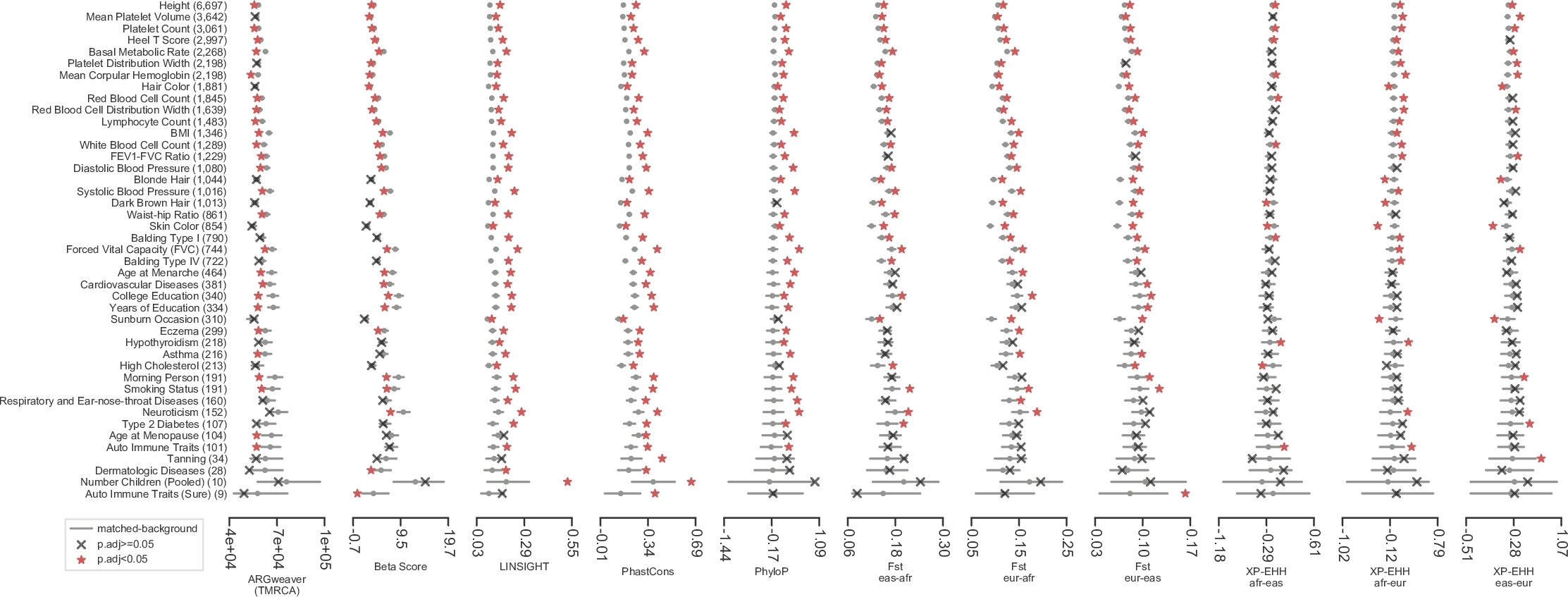

Supplement: S3 Fig — On a subset of 47 GWASs (y-axis, BOLT-LMM set), the trait-level average (red star or gray ‘x’) for 11 evolutionary measures (x-axis) compared to its matched background distribution (gray dots: mean values, gray bars: 5th, 95th percentiles) are displayed. The number of trait-associated regions is provided in parentheses. Red stars (p.adj<0.05) represent statistically significant deviation after multiple testing correction (Methods). This figure extends Fig 3A by including all 11 evolutionary measures considered in this study. (TIF) [file pgen.1010494.s004.tif]

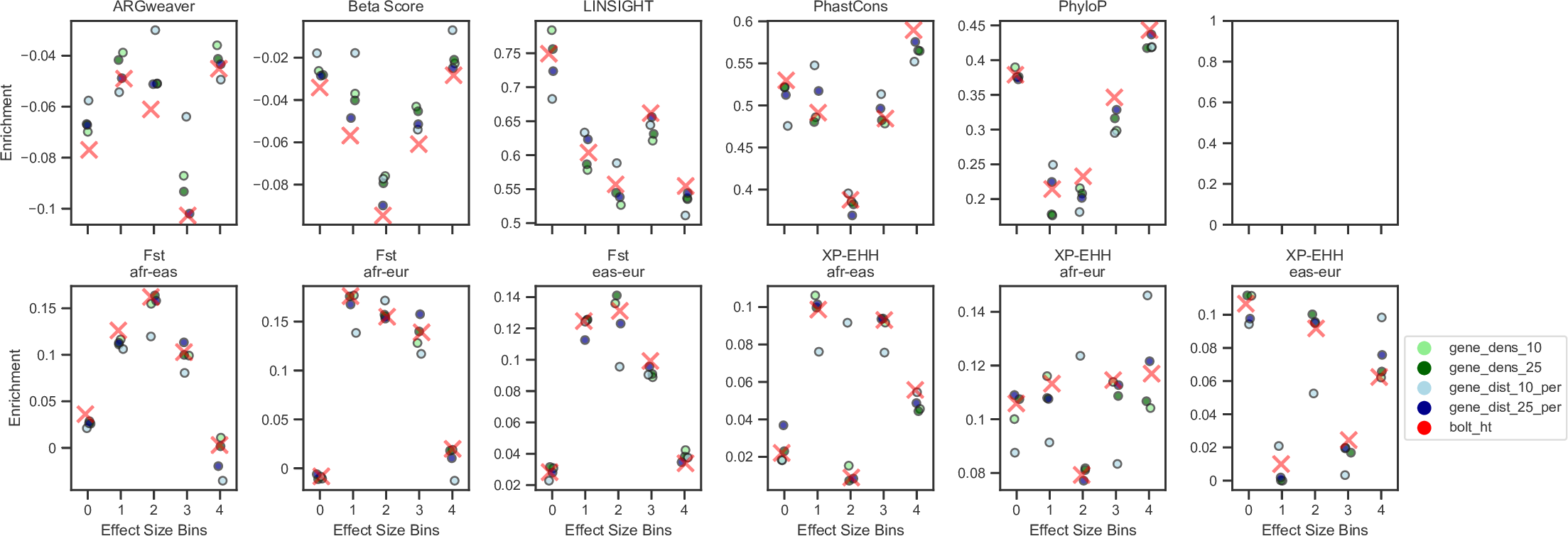

Supplement: S4 Fig — For each evolutionary measure (one plot per measure), we repeated the analysis in Fig 2B and calculated the enrichment (y-axis) across trait-associated regions partitioned by association effect size (x-axis, ordered from negative to positive effect size) for the original Fig 2B analysis (red X) and four other conditions. We repeated the analysis by changing either the gene distance matching threshold to be either +/- 25% (dark blue) or +/-10% (light blue) or the gene density matching threshold to be either +/- 25% (dark green) or +/-10% (light green) while keeping the all other parameters the same. The patterns are similar for nearly all settings. (TIF) [file pgen.1010494.s005.tif]
