# Supplementary material for: Mosaic patterns of selection in genomic regions associated with diverse human traits
Source: PLoS Genet. 2022 Nov 7;18(11):e1010494. doi: 10.1371/journal.pgen.1010494 (PMC9671423; doi:10.1371/journal.pgen.1010494)
Supplement: Attachment — Submitted filename: PGENETICS-D-22-00670_Response_updated.docx [file pgen.1010494.s007.docx]

**PLOS Genetics Reviewer Comments**

Author comments in blue text.

**Reviewer #1:** With their work the authors aim at providing a comprehensive overview of evolutionary signatures from a number of traits targeted by GWAS available from the literature.

The effort is appreciable as an evolutionary perspective on GWAS signals can indeed provide orthogonal knowledge when interpreting the association signals.

The work is clearly written and definitely deserves to be published, however there are three assumptions that should be clarified and/or addressed for the work to be fully understandable to a non specialized reader:

1) The vast majority of GWAS hits were retrieved using West Eurasian populations. Using them to pinpoint genetic regions linked to a given loci in non European populations (e.g. East Asians and Africans) introduces the assumption that the loci "tagged" by the West Eurasian GWAS are involved in the biological processes related to a given trait in all human populations. In other words, that the biology underlying a given trait is the same across all pops. This is an assumption, and as such it should be spelled out. As an example, other papers that adopted a similar approach ( <https://www.sciencedirect.com/science/article/abs/pii/S0960982222001087> ) only investigated European populations, for which this assumption can surely be granted.

We thank the reviewer for pointing out this assumption inherent to our study. We have added text to the discussion that explicitly acknowledges this assumption. Lines 418-425

2) Even taking for granted the above assumption for all human pops, surely the effect sizes (discovered in West Eurasians) were shown to be not fully transferable across pops (see Martin et al. 2017 and other relevant papers). It follows that Figure 2b (and related findings) for non European populations ( Afr-Eas, but also Eur-Eas and Afr-Eur pairs, for examples) may leverage on a weak assumption (e.g. that binning by effect sizes would yield the same binning in all the tested pops);

We agree with the reviewer. We have added text in the Discussion section [Lines 418-425] clearly stating this assumption and the evolutionary measures most likely to be affected. We have cited the Martin et al. 2017 paper as well.

3) The result that highly polygenic traits such as height are enriched for signatures of negative selection make me wonder how well "gene content" was controlled for when creating the matched-sets. I see you have a +/- 500% parameters in your recipe: perhaps you could try whether the negative selection enrichment holds when using a more conservative 200% (or some other type of matching: <https://www.cell.com/ajhg/fulltext/S0002-9297(13)00127-4)?> Or would this leave you with little or none matched SNPs? I agree that finding a matched set for a highly polygenic trait is hard; on the other hand the report of negative selection should be weighted carefully (or toned down if you cannot be sure whether you properly controlled for gene content) to avoid giving the impression that these traits are indeed under negative selection.

We thank the reviewer for raising this important point. Our matching algorithm’s criterion was +/- 50% (and not + / - 500%) for both gene density and distance to the nearest gene. We apologize for this error in the text. Regardless, we further examined the effect of matching thresholds on our final ‘enrichment’ value.

In brief, we conducted the same analysis, but modified either the gene distance matching threshold to be either +/- 25% or +/-10% or the gene density matching threshold to be either +/- 25% or +/-10% while keeping the rest of the parameters the same. We then plotted the enrichment for these four thresholds for each evolutionary measure and compared them to the enrichment observed in our original analysis (which used +/- 50% for gene distance and density). We added this figure as Fig S4 and added text in our methods section [Lines 525-531]. Even when the matching thresholds are varied (circles), the enrichment does not vary substantially compared to our original (red “X”) threshold of +/-50% across evolutionary measures, including those for negative selection, and partitioned bins based on GWAS effect size (Fig S4). These results provide support that our enrichment calculation is not sensitive to matching parameters (in this matching threshold range).

Minor points:

1) Line 61: negative selection decreases the apparent substitution rate, but I am not quite sure positive selection increases it.

We agree and have revised the text accordingly [Lines 81-84].

2) Figure 2b: I might have missed it, but could you comment more on the U shaped pattern of PhyloP? Is the U shape expected, or is it quite promising even though there is no difference in enrichment.

We thank the reviewer for asking for this clarification. We have updated the text in the results section to state that the ‘U’ shaped pattern of stronger conservation at sites with large effect size (whether trait increasing or decreasing) is expected since conservation usually indicates function and negative selection [Lines 215-217].

**Reviewer #2:** In this manuscript, Abraham et al. have generated wide-ranging and valuable resource: no less than 11 measures of natural selection calculated for over 900 GWAS. Subsequent analyses focus on general patterns that are observed for 290 high power GWAS. Although there is some risk of this study feeling like everything but the kitchen sink was included - the authors managed to pull it off and write a coherent paper. This manuscript is well written, and the multi-panel figures manage to convey a large amount of information in a concise way. Reasonable LD pruning and p-value thresholds were used for inclusion of trait-associated SNPs (the former is particularly important given that GWAS hits need not be causal SNPs). The authors also appear to have correctly implemented selection scans for each of the 11 evolutionary measures. This manuscript fits well within the scope of PLoS Genetics and is likely to be of interest to a broad readership. However, some additional analyses may be needed before this manuscript would be acceptable for publication.

One key issue is that the authors adopted outlier approaches. While there is nothing inherently wrong with this, I wonder about how many of the signatures of selection could be false positives (especially since a modest adjusted p-value cutoff of 0.05 was used). How to get around this? One option would be to use a more stringent adjusted p-value cutoff. A second option would be to use SLiM computer simulations that incorporate reasonable demographic models, and testing how each of the 11 different evolutionary measures perform. This latter option would take a lot more work to implement - and I would definitely be satisfied if the authors simply chose the first option.

We thank the reviewer for raising this important point. Some of the associations between traits and signatures of selection could indeed be false positives; however, we adjusted the nominal p-values using the Benjamini-Hochberg procedure to control the overall false discovery rate at 5%. The corresponding adjusted p-value threshold is used for each analysis, denoted in figures as p.adj, and described in the methods section (Lines 497-498**,** 555-557**,** 566-567**,** 575-577). Given the large numbers of GWASs and evolutionary measures considered and the expectation that there would be many true associations in our dataset, we opted not to use a strict family-wise error rate control like the Bonferroni correction because the likelihood of losing true signals far outweighed the risk of a small fraction of false positives. Moreover, many of the associations have adjusted p-values far below 0.05.

We agree that a simulation-based approach would better calibrate the false positivity rate for each evolutionary measure but believe it is outside the scope of this manuscript. We thank the author for this suggestion and will consider this analysis for a follow-up study.

A second key issue involves the inclusion (or lack thereof) of effect sizes. This manuscript would be greatly improved if the authors would test how well their z-scores (what the authors call "evolutionary enrichment values") are robust to including weightings of trait-specific effect sizes - i.e., relaxing point (1) on line 104. Although the authors correct that stratification and ascertainment bias is a possible confounding factor, it is important to keep in mind that some traits have genetic architectures that are driven by a small number of loci of large effect, and any tests of selection should take this into account.

We thank the reviewer for highlighting the importance of polygenicity and magnitude of effect size in contributing to the genetic architecture of a trait. We agree that effect size should not be completely ignored since the type of genetic architecture will also influence the role of selection on traits.

To balance effect size as a confounder leading to ascertainment bias while also evaluating its role on our evolutionary enrichment values, we performed our analysis on trait-associated regions after first stratifying them based on their effect size (Figure 2B). In this approach, the effect size is not directly incorporated in calculating the evolutionary enrichment value and we can assess how the enrichment value varies according to the effect size. However, we agree that the results shown in Figure 2b are based on a highly polygenic trait and the trends we observe may not generalize to non-polygenic traits. We add this as a limitation of this study and suggest this as a future approach in the discussion of our manuscript [lines 427-441]

Additional points:

1. The authors should be a bit more explicit in the sources of GWAS results, including criteria for inclusion of a particular GWAS. How many of the 290 well-powered GWAS for quantitative traits and how many are for discrete or binary traits?

We thank the author for the suggestion to clarify the source of the GWASs used in our study. We have re-written the methods section of the paper such that it now explicitly denotes the inclusion and exclusion criteria for GWASs in our evolutionary atlas (Lines 541-549). Additionally, we have added a sentence that includes the number of continuous (n=231) and discrete (n=59) traits that make up the 290 well powered GWASs (Lines 244-245).

2. Line 81: The passage that includes "the genomic background does not provide an appropriate null when interpreting overlaps between trait associations and signatures of selection" is a little unclear especially since line 105 includes the phrase "builds a null distribution from allele frequency and LD-matched SNPs." The authors are advised revise for clarity.

We thank the reviewer for pointing out this unclear phrasing. We have modified the sentence to more explicitly state that the genomic background in this context is referring to averaging across all variants in the human genome and that matching on factors relevant to selection and association like LD and frequency is necessary (Lines 103-104).

3. "Evolutionary enrichment" seems a little bit of a misnomer. tThat said, the approach used by the authors yields a reasonable summary statistics. I wonder if "Evolutionary z-score" might be a better way of writing things.

We thank the reviewer for raising this point regarding the term ‘enrichment.’ The intuition behind our enrichment measure is to capture a fold difference in an evolutionary metric when an appropriately matched background is used. That said, we agree that the definition of ‘evolutionary enrichment’ may not be immediately obvious to a reader. To mitigate confusion, we took extra care to be consistent throughout the manuscript and explicitly denote ‘positive’ vs. ‘negative’ enrichment corresponding to positive and negative values of our evolutionary enrichment statistic where necessary. Additionally, we have added a sentence within the first results section to explicitly acknowledge that enrichment may be positive or negative (Lines 155-156).

We appreciate the reviewer’s idea of labeling the summary statistic as an ‘evolutionary z-score.’ Indeed, the evolutionary enrichment we measure is very similar to a traditional z-score. For a traditional z-score, the denominator is the standard deviation of the distribution. For the evolutionary enrichment as we have defined it, the denominator is a standard deviation of the evolutionary measure across the genome, which is different from the empirical background distribution generated from the matched sets that we use to compare the trait-associated regions mean. Although this is a subtle distinction, we believe that our evolutionary enrichment is not the same as a true z-score. Thus, we have kept the term ‘evolutionary enrichment’ because it broadly captures the intuition of a fold difference and do not want to introduce the potential for this confusion about the meaning of a ‘z-score’.

4. I like that their approach was tested on height. However this is an exceptionally polygenic trait, (and therefore less likely to be affected by the decision to not directly incorporate effect sizes - as per line 104).

The reviewer raises an important observation that height is a very polygenic trait. We agree that the polygenicity of height makes our observations for this trait more robust to not incorporating effect sizes. We address this as a limitation of our approach and suggest that this is an interesting topic worthy of future careful examination [**lines 494-508**].

5. Table 1: It would be advisable to list the actual time scale (in terms of ky or My for each of these evolutionary measures. Also, "Evolutionary origin" seems like an odd term here. Maybe change it to something like TMRCA and keep ARGweaver in the first column?

Thank you for this suggestion. We have updated the table with the approximate timeframe for each evolutionary measure and removed ‘evolutionary origin’ for ARGweaver (Lines 785-793).

6. Figure 2b: It seems overly redundant to partition things based on negative and positive effect size (as evidenced by the symmetry of each of the subplots). It makes much more sense to partition using the absolute value of effect size (perhaps at finer resolution to retain five bins per evolutionary measure). Alternatively, the authors could partition trait-associated SNPs by p-value or MAF.

We thank the reviewer for this suggestion of using absolute values of the effect size for partitioning in Figure 2b. We agree that many of the trends we observe are symmetric and therefore an absolute value-based partition would also highlight the same trend. We choose to present these data with the sign of the effect to explicitly illustrate this symmetry and maximize its clarity for a general audience. We have provided a partitioning by p-value for Figure 2b as a supplemental figure (Supplemental Figure 2) and described the results in lines 222-225.

7. Figure 3b: enrichment scores of -1 or +1 are still pretty modest. Why doesn't the enrichment score need to be > |2| for things to be significant?

We agree that the enrichment scores in Figure 3b are modest but still informative in illustrating different evolutionary profiles across the evolutionary measures for different traits. Many of the distributions of the statistics we consider are not normal, and thus using a threshold greater than |2| to define statistical significance or convert from z-scores into p-values is not possible. To address this, we empirically generated matched background distributions for each trait and evolutionary measure. (And indeed, many of these distributions are not normal.) For example, for measures like Fst, where the genome average is very close to zero with very few variants having high Fst values, the empirically generated matched background distribution will not be normal but instead rather skewed. In skewed distributions, interpretation of z-scores is not straightforward. With this in mind, in this study we calculate an empirical p-value by counting the fraction of the matched background sets that are as or more extreme than the observed trait-associated value. We then adjust this nominal p-value for multiple testing using the Benjamini Hochberg procedure to control the false discovery rate at 5% to ensure we have a stringent criterion for statistical significance.

8. Line 353: Great hypothesis. Why not explicitly test whether genomics regions that are associated with late-onset traits are less likely to have strong signatures of selecting with the data that is in hand? Adding a small analysis along these lines would improve this paper.

We agree that a comprehensive analysis of early and late-onset traits would help resolve this question. Ideally, we would compare Alzheimer’s disease to other late-onset neurological diseases. However, there were no other late-onset neurological traits that met our cutoff of 50 GWAS-associated regions. We do, however, have three different GWAS for Coronary Artery Disease (CAD). Within these three GWASs, we see a consistent enrichment in values for LINSIGHT, phastCons, and phyloP. Two of the three GWAS also had enrichment in Fst between individuals of European and either East Asian or African ancestry. One GWAS (conducted in the UK BioBank samples) also had depletion in ARGweaver, allele age. Thus, the lack of strong signatures seems specific to late-onset Alzheimer’s, but this will require further comparisons on GWAS curated to answer this question. We have updated the text [306-313] to reflect these additional data.

**Reviewer #3:** Abraham & LaBella et al develop a new framework for analyzing the signals of selection underlying complex trait-associated variation. They apply this method to human GWAS summary statistics and genomic data and describe several findings pertaining to differences between associated regions and background control regions for several statistics such as Fst, XP-EHH, and PhyloP.

I found the manuscript quite interesting, and it has several strengths, including a novel framework for sampling genomic background regions that could prove useful in future research, some interesting observations about potential evolutionary mechanisms that might underly the enrichments/depletions they detect, and an appreciated review of some limitations in the discussion section. I could see myself using their approach, especially paired with model-based simulations to explore the effects of evolutionary processes on the joint distribution of these statistics.

I do have some comments about the interpretation of the observations in this manuscript. In general, the authors have described this method as a way to characterize evolutionary processes underlying trait variation, and they have categorized the various statistics they use (e.g., Fst) by specific evolutionary processes (e.g., positive selection). However, most evolutionary processes (different modes of selection, demographic processes, recombination, mutation rate changes, etc) will have some impact on these statistics, and hence it is difficult for me to see how differences in the distributions between background and foreground sets could be ascribed directly to specific processes.

Differences between foreground and background sets can be described as consistent with the action of various selection modes (e.g., differences in Fst are consistent with positive selection), but other evolutionary processes usually cannot be ruled out based on this type of analysis. To me this is a very important distinction, related to several ongoing debates in the field about the relative impact of various evolutionary processes on genomic variation. I provide a few specific examples below, followed by some minor comments. I think this main concern could be addressed primarily by changing the wording throughout the manuscript. The method could be described as an approach for rigorous assessment of enrichment/depletion of evolutionary patterns, possibly driven by various evolutionary processes.

Main comments:

As I’m sure the authors are aware, multiple different evolutionary processes could affect the summary statistics that they have included in their analysis. I’m going to briefly itemize some studies that specifically address how these statistics are affected by processes that could differ between background and foreground sets.

1. Haplotype scans may be affected by demographic processes and multiple modes of selection (Tadashi et al 2006 Genome Research). While the whole genome experiences the same demography in a panmictic population, the same is not necessarily true in a real GWAS sample with any mixed ancestry or admixed individuals. (Variation in background selection will also cause the effects of demography to be experienced differently by different parts of the genome, as in Torres 2018, noted below). XP-EHH differences might be affected by such processes and not only due to positive selection.

2. Fst is affected by both background selection (Torres et al 2018 Plos Genetics) and mathematical constraints on the values it can take (Jakobsson et al 2013 Genetics). Fst can be elevated under positive selection, but causal SNPs may also be excessively differentiated under other selection modes (e.g., stabilizing selection w/o any change in the mean, Yair & Coop, Phil Trans B 2022).

3. Balancing selection is likely to generate high diversity and short haplotypes, and hence is very likely to affect several of the statistics used here. Fst has been used to detect balancing selection amongst other modes of selection (Foll & Gagiotti 2008 Genetics; see also Ecxcoffier 2009 Heredity) under the principle that allele frequencies should be more similar than expected. However, if the environment changes but balancing selection is maintained, the equilibrium frequency of the balanced allele will change, resulting in unknown (and unpredictable) effects on Fst.

Point 1) These are just some examples — in general I don’t think it’s possible to directly ascribe an evolutionary process to differences in the distributions of these statistics. The authors could revise the manuscript by clarifying how their findings are consistent with various modes of selection, rather than fully explained by these evolutionary processes. They could also include a short section describing the ways that multiple evolutionary processes could underly some of the differences in these statistics in the discussion.

We completely agree with the reviewer and thank them for the references. Our analyses characterize patterns of statistics that are each influenced by multiple evolutionary processes. For simplicity, we linked these statistics with specific evolutionary processes, but we agree that this could give the incorrect impression that there is a one-to-one correspondence. We have clarified our language to not directly equate the statistics to modes of selection throughout the manuscript. We also have added text on the ways that multiple processes can shape these statistics in the Discussion [338-342].

Point 2) In addition, I think that the finding that effect sizes are correlated with the values of some statistics could be further justified (Fig 2b). As I’m sure the authors are well aware, effect size and frequency are major factors in determining the power to detect associations. In the absence of any selection-driven relationship between effect size and frequency, lower frequency GWAS alleles tend to have bigger effects (Park et al 2011 PNAS, “Distribution of allele frequencies …”). Allele frequency is also a major determinant of Fst — low frequency alleles are more constrained in terms of the values of Fst that they can obtain (see the Jakobsson citation above). This could result in spurious correlations between effect sizes and Fst, or any other statistic whose values are partially determined by allele frequency.

We thank the reviewer for raising this important point. We agree that effect size, allele frequency, and power to detect variants with GWAS influence one another and many of the evolutionary statistics considered. This was the motivation for generating background sets matched on LD and allele frequency. Thus, the relationships between each statistic and effect size (as in Figure 2b) are based on comparisons with background sets that have similar distributions. Nonetheless, we agree that fully accounting for these relationships is challenging and have added discussion of these points and limitations [Lines 397-404].

Minor comments:

1. Methods: Can the authors clarify how they have performed the LD matching? I could not follow the details in the manuscript. It seems like they have pruned on r^2, but this is a pairwise metric and it was unclear to me how they extend it to a genomic region. Do they match the LD spectrum? Did they consider other methods to match LD, such as matching on LD score?

We thank the reviewer for asking for clarification on the matching and LD expansion. We have updated the methods section to further clarify (Lines 460-469). Briefly, for a given trait-associated region, the number of trait-associated variants in LD (>0.9) with the lead trait-associated variant is counted (number of LD “buddies”). We then identify matched control variants based on their frequency and a similar number of variants in LD with the matched control variant.

2. Methods: How do the authors ensure that there are enough matched genomic background regions to provide a good null? In particular, when using 500kb windows, there are only ~6000 such windows in the genome. If a GWAS has several hundred independent hits, are there enough windows remaining that match on LD and frequency such that the different random background samples are actually independent, and not formed repeatedly from a small subset of windows? The tests could be anti-conservative if the set of windows making of the background are highly correlated across each of the 5,000 samples, resulting in correlations between each set that do not reflect the true variance.

We thank the reviewer for pointing out this unclear point. While we allowed the associated region to expand up to a 500 kb window, the regions were significantly smaller than that. For example, the median matched region size for the Height GWAS was ~15 kb and the mean was 34 kb.

3. Abstract: "was the most dominant", this seems ambiguous please clarify.

We have modified the text to say ‘included’ instead of ‘most dominant’ in the abstract (Lines 39-40). We thank the reviewer for pointing out this ambiguity.

4. Abstract: is “negative enrichment” the same as “depletion”? Perhaps depletion would be clearer.

We thank the reviewer for raising this question. Yes, negative enrichment is the same as depletion. For the sake of consistency, we have used “positive” and “negative” enrichment throughout the manuscript. We have also added text making this definition explicit [Lines 155-157].

5. The first paragraph makes it sound like selection is known to be the most important evolutionary process affecting human differentiation. Drift is also undoubtedly very important. I think any group of population geneticists could argue unproductively over the specifics for hours, but the intro should make it clear that drift has a substantial role and exactly how much differentiation is fitness-related and how much is neutral is not known.

We agree and have added reference to the influence of non-selective forces in contributing to modern genetic architecture for complex traits in the first paragraph (Lines 68-70).

6. Line 94: "Despite these complications..." This makes it sound like these studies have gotten around the stratification issue to find robust selection evidence. I agree that some polygenic selection findings are more robust than others, but I'm not aware of any that completely remove any possible confounding. If the authors disagree they should provide a reason, as I do not think this is clear (at least to me).

We agree and thank the reviewer for making his nuance explicit. We just meant to indicate that some progress has been made. We have modified the sentence to draw attention that certain traits have consistently shown signatures of selection across studies with diverse methodological approaches. [Lines 103-104]

7. Line 101-102: It’s true that many previous papers have been motivated by understanding the processes that could shape traits, but many have been clear that we cannot distinguish between selection on the traits and selection on trait variation.

We agree with the reviewer and have deleted the phrase “that focused on traits themselves” from this sentence. [Lines 117-121]

8. Line 424: “A second major difference is that we do not directly consider effect size or direction inferred 425 from GWAS”, I agree with the author’s point in this section that this can avoid some issues with stratification. However, alleles near the threshold for detection (alleles with small effect sizes that barely pass the threshold for significance) may still be stratified because they are more likely to be false positives. Some previous methods have also used only the sign of the effect size and not the magnitude (e.g. Turchin et al).

Yes, we agree as well. We have modified the sentence to state this particular case (Lines 377-380).

Thanks for the opportunity to review your work!

Sincerely,

Lawrence Uricchio
